# Supplementary material for: A New Method for a Polyethersulfone-Based Dopamine-Graphene (xGnP-DA/PES) Nanocomposite Membrane in Low/Ultra-Low Pressure Reverse Osmosis (L/ULPRO) Desalination
Source: Membranes (Basel). 2020 Dec 18;10(12):439. doi: 10.3390/membranes10120439 (PMC7766060; doi:10.3390/membranes10120439)
Supplement: Supplementary file 1 [file membranes-10-00439-s001.pdf]

Supplementary Materials

A New Method for a Polyethersulfone-based Dopamine-Graphene (xGnP-DA/PES) Nanocomposite Membrane in Low/Ultra-low Pressure Reverse Osmosis (L/ULPRO) Desalination

Lwazi Ndlwana [1,*], Mxolisi M. Motsa [1], and Bhekie B. Mamba [1,2]

1. Institute for Nanotechnology and Water Sustainability Research, College of Science, Engineering and Technology, University of South Africa, Florida Science Campus Florida, Johannesburg 1709, South Africa; ndlwal@unisa.ac.za (L.N); motsamm@unisa.ac.za (M.M.M)
2. School of Materials Science and Engineering, Tianjin Polytechnic University, Tianjin 300387, PR China; mambabb@unisa.ac.za

* Correspondence: ndlwal@unisa.ac.za; Tel.: (+27-82-591-4864)

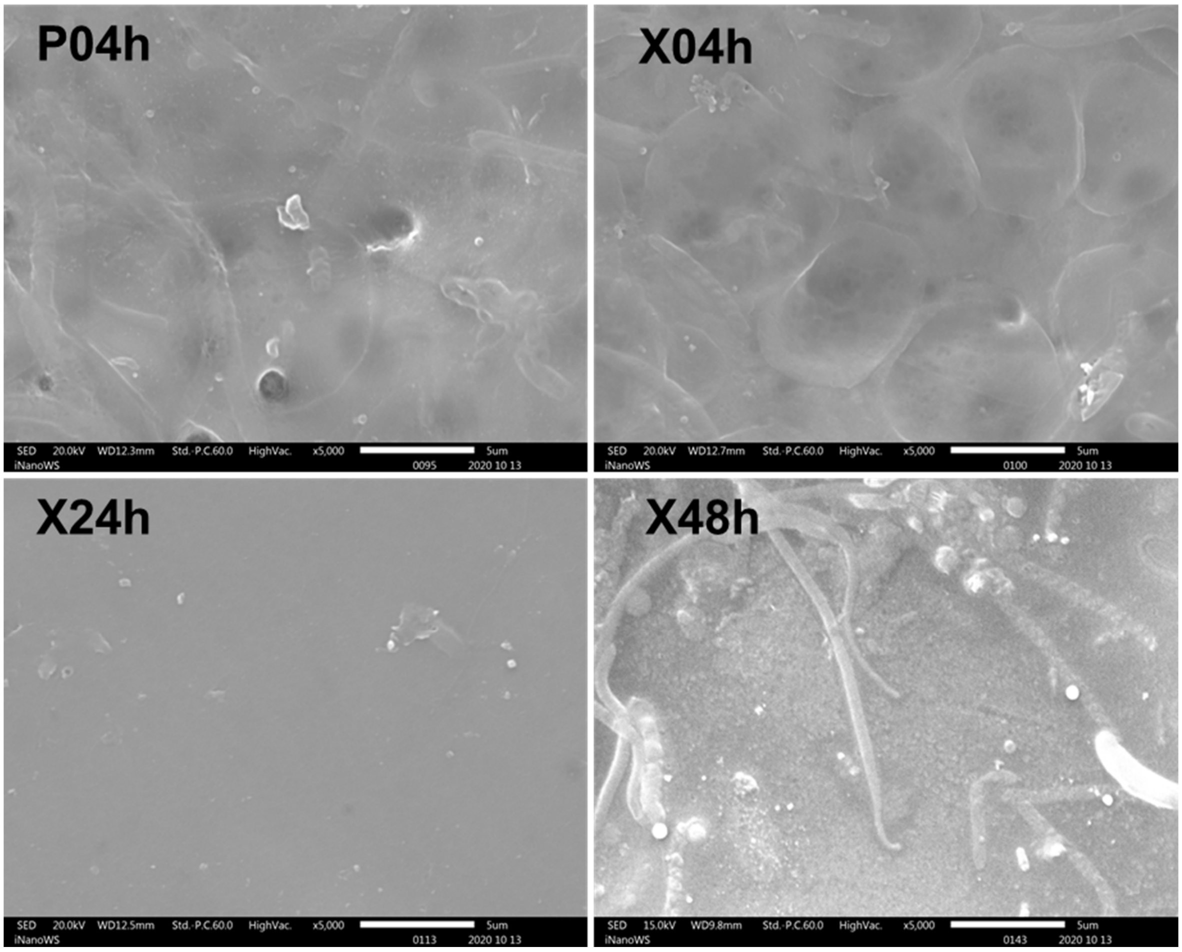

Figure S1. Higher magnification surface SEM images for the membranes prepared, depicting the change in surface morphology due to the modification and fabrication method.
